# Reduced attentional inhibition for peripheral distractors of angry faces under central perceptual load in deaf individuals: evidence from an event-related potentials study

**DOI:** 10.3389/fnhum.2023.1162488

**Published:** 2023-08-17

**Authors:** Jun Huang, Linhui Yang, Kuiliang Li, Yaling Li, Lan Dai, Tao Wang

**Affiliations:** ^1^School of Education Science, Chongqing Normal University, Chongqing, China; ^2^Chongqing Key Laboratory of Psychological Diagnosis and Education Technology for Children With Special Needs, Chongqing, China; ^3^Changsha Special Education School, Changsha, China; ^4^Banan Special Education School, Chongqing, China

**Keywords:** deaf, perceptual load, peripheral vision, angry face, ERPs, P1, N170

## Abstract

**Background:**

Studies have shown that deaf individuals distribute more attention to the peripheral visual field and exhibit enhanced visual processing for peripheral stimuli relative to hearing individuals. This leads to better detection of peripheral target motion and simple static stimuli in hearing individuals. However, when threatening faces that represent dangerous signals appear as non-targets in the periphery, it remains unclear whether deaf individuals would retain an advantage over hearing individuals in detecting them.

**Methods:**

In this study, 23 deaf and 28 hearing college students were included. A modified perceptual load paradigm and event-related potentials (ERPs) were adopted. In the task, participants were instructed to search for a target letter in a central letter array, while task-irrelevant face distractors (happy, neutral, and angry faces) were simultaneously presented in the periphery while the central perceptual load was manipulated.

**Results:**

Behavioral data showed that angry faces slowed deaf participants' responses to the target while facilitating the responses of hearing participants. At the electrophysiological level, we found modulation of P1 amplitude by central load only in hearing individuals. Interestingly, larger interference from angry face distractors was associated with higher P1 differential amplitude only in deaf individuals. Additionally, the amplitude of N170 for happy face distractors was smaller than that for angry and neutral face distractors in deaf participants.

**Conclusion:**

The present data demonstrates that, despite being under central perceptual load, deaf individuals exhibit less attentional inhibition to peripheral, goal-irrelevant angry faces than hearing individuals. The result may reflect a compensatory mechanism in which, in the absence of auditory alertness to danger, the detection of visually threatening information outside of the current attentional focus has a high priority.

## Introduction

Early auditory deprivation can attenuate individuals' ability to process auditory information, such as an inability to detect environmental sounds and recognize human speech, and may be typically accompanied by deficits in cognitive, emotional, and social function if not promptly corrected (Arlinger, 2003). However, it has also been shown that early auditory deprivation may improve visual processing, a phenomenon known as cross-modal plasticity (Lee et al., 2001; Bavelier and Neville, 2002). The most commonly reported finding has been that deaf individuals possess better peripheral vision compared to hearing individuals. Specifically, compared to hearing individuals, deaf individuals have demonstrated superior detection of peripheral motion targets and a stronger neural response related to attention and motion processing (Neville and Lawson, 1987; Bavelier et al., 2000; Shiell et al., 2014). In addition, substantial research has reported that deaf individuals show an enhanced distribution of attention toward peripheral spatial locations compared to hearing individuals (Proksch and Bavelier, 2002; Sladen et al., 2005; Bavelier et al., 2006; Dye et al., 2007; Chen et al., 2010). In these studies, the spatial distribution of deaf individuals' attention has been typically measured using a modified perceptual load paradigm (Lavie, 1995). In the task, target geometrical shapes are presented in the center of the visual field, while incongruent geometrical shape distractors are presented in the central or peripheral visual field. Participants are required to detect the centrally prescribed target and ignore the irrelevant distractor, while the central perceptual load is varied. It has been reported that hearing individuals' responses to central targets are more affected by central distractors, whereas deaf individuals are more affected by peripheral distractors (Proksch and Bavelier, 2002; Bavelier et al., 2006; Chen et al., 2010). Intriguingly, increased attentional spread to peripheral locations in deaf individuals has been shown to be especially pronounced when attentional resources are limited (Proksch and Bavelier, 2002; Bavelier et al., 2006; Hauthal et al., 2012). Proksch and Bavelier (2002), for instance, found that when central perceptual load increased, interference from peripheral geometrical shapes was eliminated earlier in hearing individuals than in deaf individuals, a result that was proposed to reflect greater attentional capacity in the peripheral visual field of deaf individuals. Some researchers have argued that the altered spatial distribution of visual attention in deaf individuals is caused by deafness *per se*, rather than extensive experience with sign language (Proksch and Bavelier, 2002; Bavelier et al., 2006) and likely reflects a visual compensation for the lack of auditory alertness to stimuli outside the current attentional focus (Bavelier et al., 2006; Chen et al., 2010). At the neural level, neuroimaging studies have shown that improved peripheral visual skills in deaf people involve the activation of multiple systems, including the primary auditory cortex, supramodal, and multisensory regions (Scott et al., 2014; Seymour et al., 2017).

In addition to meaningless stimuli (e.g., geometrical shapes), the visual attention and processing of highly meaningful stimuli present in the periphery have also been recently investigated, albeit sparsely. Faces are socially and biologically significant stimuli that draw attention more than non-face objects (Langton et al., 2008). For example, Hauthal et al. (2012) used male and female symbols as targets and three non-gender object symbols as fillers, forming a virtual square shape around the center as a judging array. As distractors, neutral female or male faces were shown in peripheral or central locations. Participants were instructed to classify the gender of the target symbol. The target and distractor were either gender-congruent or gender-incongruent, and the extent to which distractors captured participants' attention was indexed by the size of gender congruency effects. Results of this study found that, regardless of the location of the face distractors, gender congruency effects in hearing participants only existed under low perceptual load, whereas the effects in deaf participants were not impacted by perceptual load. This result suggests that when central load increases, hearing individuals' attentional capacity exhausts, whereas deaf participants appear to retain free capacity to detect faces in the periphery, which is consistent with previous findings indicating that deaf individuals possess greater attentional resources in the periphery than hearing individuals. In contrast, Shalev et al. (2020) presented peripheral faces as targets and required participants to identify a variety of facial characteristics. Interestingly, they did not find that deaf participants had superior perceptual representations for face identification, gender, or eye gaze direction; however, they did observe that deaf participants outperformed hearing participants in recognizing facial expressions, particularly fearful expressions. Similarly, a recent study by Lee et al. (2022) reported that deaf individuals perform better in the peripheral facial discrimination task than hearing individuals. Taken together, these results suggest that deaf people's enhanced peripheral visual attention and processing also apply to stimuli that are more complex and meaningful.

Based on previous studies (Proksch and Bavelier, 2002; Hauthal et al., 2012; Shalev et al., 2020), this study investigated whether the advantage for deaf individuals' attention to peripheral facial expressions was maintained when faces were presented as non-targets. Expressions are particularly salient facial features, carrying important psychological and biological information (Itier and Neath-Tavares, 2017). Emotional expressions, especially threatening expressions (e.g., angry expressions), can automatically capture individuals' attention and receive priority processing, even when presented as non-targets (West et al., 2009). This process was thought to be adaptive because threat expression represents a dangerous signal, and rapid detection of it can improve survival chances (Fox et al., 2000). In fact, facial expression is a more prominent stimulus for deaf individuals when compared to hearing individuals, in terms of both social interaction and language (Mitchell and Maslin, 2007). Due to auditory deprivation, deaf individuals rely heavily on facial expressions to rapidly obtain emotional and social information that hearing individuals typically receive through tone of voice (Mitchell and Maslin, 2007). Furthermore, sign language is the primary mode of communication for the majority of deaf people, and facial expressions are crucial semantic and grammatical components for the comprehension of sign language (Elliott and Jacobs, 2013). Consequently, when facial expressions appear as non-targets in the periphery, automatic and accurate detection of them should play a crucial role in the social interaction and survival of deaf people.

Given that enhanced peripheral visual attention in deaf people is best revealed under attentional demanding conditions (Hauthal et al., 2012), a modified version of Lavie's (2005) perceptual load paradigm was used in this study. In this task, the target is presented in the central visual field and a face distractor is presented in the peripheral visual field, while the central perceptual load is varied by manipulating the discrimination difficulty of the target. According to perceptual load theory (Lavie, 1995), when the perceptual load of the central task is low and does not exhaust cognitive resources, the remaining resources are spared to process distractors involuntarily, which can interfere with the task by causing longer reaction times (RTs). However, individuals' cognitive capacity is limited; when the perceptual load is increased, the interference from distractors is reduced or even eliminated. Therefore, the size of interference effects can index the attentional resources allocated to peripheral face distractors (Lavie, 2005). This study synchronously recorded event-related potentials (ERPs) to uncover the time course of attention to facial expressions due to their extremely high temporal resolution (Hillyard and Anllo-Vento, 1998).

Previous studies utilizing ERPs have found that two early ERP components, P1 and N170, are sensitive to the emotional content of facial expressions within 200 ms after face presentation (Luo et al., 2010; Rellecke et al., 2012; Schindler and Bublatzky, 2020). The P1 appears between 80 and 130 ms after stimulus onset at occipital sites and is assumed to be generated within the extrastriate visual cortex (Itier and Neath-Tavares, 2017). The mediation of P1 by facial expressions is thought to be driven by low-level visual cues, such as spatial frequency and contrast, rather than emotional representation (Itier and Neath-Tavares, 2017). The face-sensitive N170 is recorded from occipito-temporal electrodes 130–200 ms post-face onset, with its neural origins including the fusiform gyrus, the inferior occipital gyrus, and even possibly the superior temporal sulcus, which is thought to reflect higher-level structural encoding of faces (Itier and Neath-Tavares, 2017). It has been demonstrated that emotional information from face stimuli is preferentially differentiated in central rather than peripheral vision (Holmes et al., 2006). For example, when a face is presented in the foveal vision, emotional faces, especially those that are threat-related (e.g., angry faces), produce a larger P1 or N170 than neutral faces (Batty and Taylor, 2003; Luo et al., 2010; Hinojosa et al., 2015). Moreover, many studies have indicated that these emotional effects of P1 or N170 seem to be independent of attentional resources (Itier and Neath-Tavares, 2017; Luo et al., 2017; Schindler et al., 2021). However, when faces appeared in peripheral vision, most research has consistently reported that the emotional effects of early ERP components, including P1 and N170, were eliminated by inattention or central perceptual load (Holmes et al., 2003; Wang et al., 2016; Schindler et al., 2022). This may be related to the fact that facial expression discrimination typically requires detailed foveal scrutiny (Levy et al., 2001), and this process is impeded by limited attentional resources and declined visual acuity in peripheral vision.

In summary, this study utilized the modified perceptual load paradigm and ERPs to investigate the attention of deaf individuals to peripheral facial expressions that appeared as non-targets. Prior research has shown that the enhanced attention to the peripheral visual field observed in deaf individuals may increase sensitivity to dangerous peripheral stimuli (Bavelier et al., 2006; Shalev et al., 2020). Therefore, deaf individuals may have an advantage over hearing individuals in detecting threatening faces in the periphery. More specifically, we predicted that interference effects produced by threatening faces would be larger in deaf individuals than hearing individuals, indicating that it is more difficult for deaf individuals to inhibit their attention to threatening face distractors than for hearing individuals. Additionally, we also predicted that deaf individuals' enlarged attention capacity in the peripheral visual field would enable them to distinguish between threatening and non-threatening faces, but that this process would be impeded in hearing individuals (Holmes et al., 2003; Wang et al., 2016). If so, threatening faces would produce more interference than non-threatening faces in deaf people, whereas this effect would be absent in hearing individuals. Importantly, these effects in deaf people may not be modulated by perceptual load. At the neurological level, we also expected to observe P1 or N170 responses that were compatible with interference effects.

## Materials and methods

### Participants

G^*^Power 3.1 was used to calculate the required sample size, with an alpha level of 0.05, a power of 0.8, and a median effect size of 0.25. According to the power analysis results, a total sample size of 30 would be required to ensure adequate statistical power. Additionally, according to similar previous studies (e.g., Hauthal et al., 2012), a total of 30 participants is enough to detect group differences. Therefore, taking the above information into account, a total of 54 participants were recruited from Chongqing Normal University in China to ensure adequate observation of experimental effects; 24 of the participants were deaf students from the Department of Special Education, and 30 matched hearing controls were recruited from the same geographic area. All deaf participants were native signers and had a hearing loss of > 71 dB. The mean age of onset of their deafness was 1.72 years, ranging from 0 to 7 years. All participants were right-handed, had normal or corrected-to-normal vision, and none had a history of neurological or psychiatric disorders. Additionally, none of the study subjects had participated in a similar experiment. One deaf and two hearing participants were excluded for excessive missing EEG data due to more than half of the trials being contaminated by slow drift artifacts or too many eye movements. Ultimately, data from 23 deaf [13 women and 10 men, mean age (*M*) = 20.80 years, standard deviation (*SD*) = 1.67, range from 18 to 23 years] and 28 hearing [18 women and 10 men, mean age (*M*) = 20.82 years, *SD* = 1.02, range from 19 to 22 years] participants were included for further analyses. The demographic information of the hearing and deaf groups is summarized in Table 1. The study received ethics clearance from the Chongqing Key Laboratory of Psychological Diagnosis and Education Technology for Children with Special Needs Research Ethics Board. All participants provided written informed consent prior to the experiment in accordance with the Declaration of Helsinki and were paid for their participation after the experiment.

**Table 1:** The demographic information for the hearing and deaf groups.

**Demographic information**	**Hearing (*N =* 28)**	**Deaf (*N =* 23)**
Mean age (years, *M* ±*SD*) and range	20.82 ± 1.02,19-22	20.80 ± 1.67,18-23
Gender	10 men, 18 women	13 men,10 women
Communication language	Oral language	Sign language
Hearing loss	Normal	>71 dB
The mean age of deafness onset and range (years)	/	1.72, 0–7

### Stimuli and procedure

**Figure 1:**
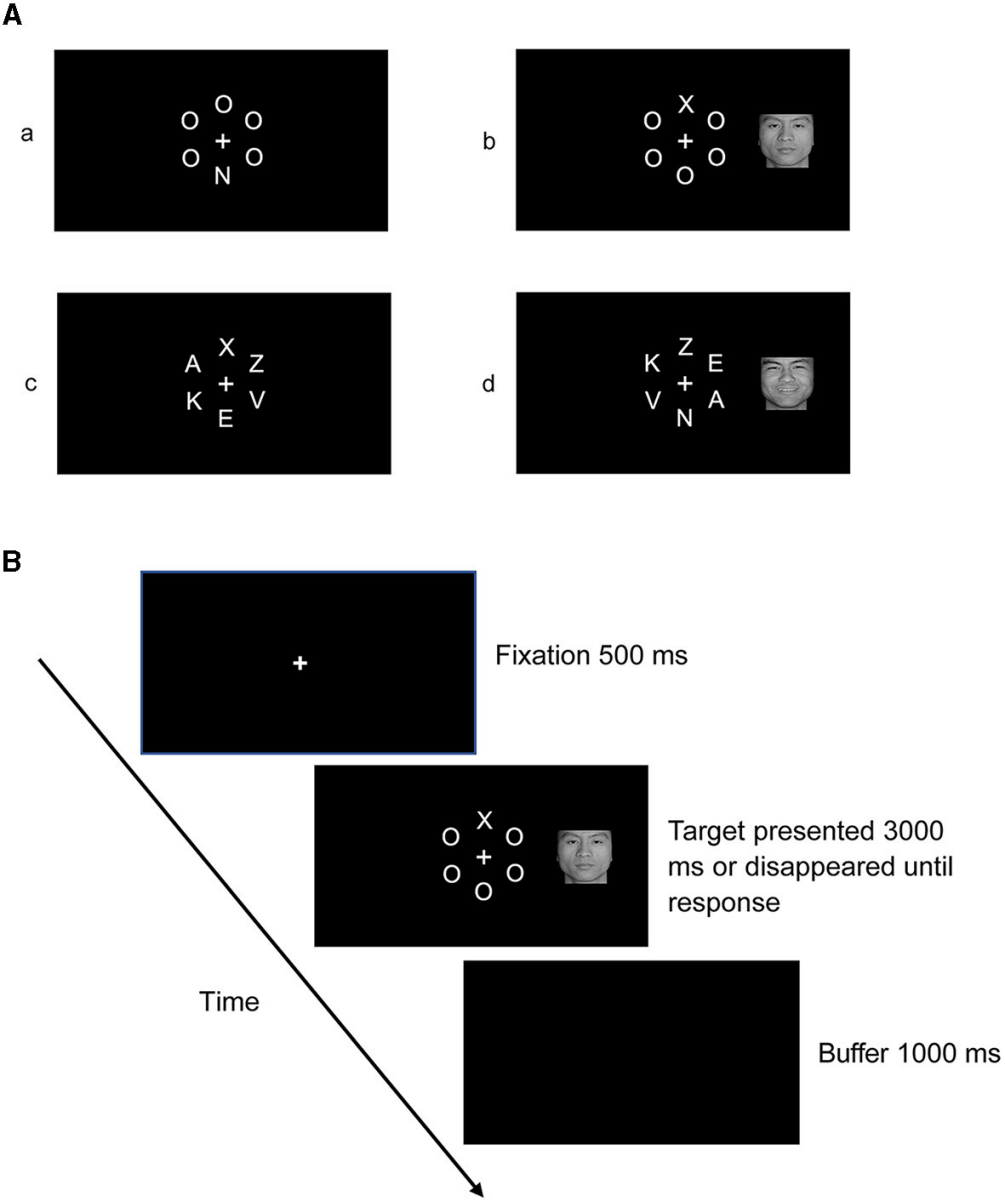
**(A)** Stimuli in each condition: (a) stimuli in the condition of no face distractor presented under low-perceptual load; (b) stimuli in the condition of the target presented with a face distractor under low-perceptual load; (c) stimuli in the condition of no face distractor presented under high-perceptual load; (d) stimuli in the condition of the target presented with a face distractor under high-perceptual load. **(B)** Schematic representation of the procedure.

Stimuli consisted of a letter array around a white fixation presented alone or with a face picture concurrently positioned to the left or right of the fixation against a black background. Figure 1A shows the schematic representation of the stimuli. In the low-perceptual load condition, arrays consisted of the target letter “X” or “N” and five “O” fillers. These fillers were presented together in the form of a virtual circle around a white fixation point in the center, with a radius of 2 cm (visual angle of 3.82°). The target letter (“X” or “N”) appeared randomly but with equal probability in one of six positions arranged in the circle at 0°, 60°, 120°, 180°, 240°, and 300°. The other five locations were occupied by the five fillers. In the high-perceptual load condition, the same stimuli were used as in the low-perceptual load condition, except that the five “O” fillers were substituted for the letters “A,” “K,” “E,” “V,” and “Z.”

A total of 48 pictures of faces were selected from the native Chinese Facial Affective Picture System (Gong et al., 2011), including 16 happy faces, 16 angry faces, and 16 neutral faces (50% ratio of female/male faces). To exclude the influence of face identity (e.g., attractiveness) on participants' attentional allocation, the individual happy, neutral, and angry expressions were selected from the same actor. These pictures of faces were processed using Photoshop into black and white photographs, keeping uniform size, luminance, and contrast. The valence and arousal of the three categories of faces were matched in terms of the normative rating scores (see Table 2). On the valence dimension (1 = highly negative, 9 = highly positive), the valence of happy faces (6.27 ± 0.40) was larger than that of neutral faces (4.40 ± 0.50, *p* < 0.001) and angry faces (2.93 ± 0.43, *p* < 0.001). On the arousal dimension (1 = highly calm, 9 = highly arousing), the happy (5.00 ± 1.21) and angry faces (5.63 ± 1.29) demonstrated no significant difference, but both had larger arousal than neutral faces (3.69 ± 0.48, *p* < 0.001).

**Table 2:** The valence and arousal of happy, neutral, and angry facial expressions (*M* ± *SD*).

**Attribution**	**Facial expression**			***F***
	**Happy**	**Neutral**	**Angry**	
Valence (*M* ±*SD*)	6.27 ± 0.40	4.40 ± 0.50	2.93 ± 0.44	224.83^***^
Arousal (*M* ±*SD*)	5.00 ± 1.21	3.69 ± 0.48	5.63 ± 1.29	13.99^***^

^***^p < 0.001.

Participants were seated in a dimly lit and sound-attenuated room; the stimuli were presented on a 17-inch Samsung display at a resolution of 1,024 × 768 pixels with a refresh rate of 75 Hz. The distance between the participant and the computer monitor was approximately 60 cm. There were 576 trials in total, consisting of eight blocks: four blocks for the low-perceptual load condition and four blocks for the high-perceptual load condition. The blocks were presented in a fixed order, and the presentation sequence was balanced between participants. Each block included 72 trials; to improve the possibility that participants' attention was captured by faces, within each block, 48 trials (accounting for 66.67%) presented a letter array alone, while 24 trials presented a letter array in the center with a face distractor (8 trials each for happy, neutral, and angry face distractors, accounting for 33.33% in total) in the left or right visual field (to keep face novelty). The visual angle of the distracting face picture's nearest border was 4.10° relative to the central fixation, and the visual angle of the distracting face picture's farthest border was 7.13° relative to the central fixation, falling within the previously defined range of the peripheral visual field (Chen et al., 2010; Pavani and Bottari, 2012). During each experimental trial, a 500-ms fixation was first presented on the center of the screen and then again when the target was presented (letter array only or with a face picture to the left or right). Participants were required to press buttons “1” for target “X” and “3” for “N” during this time interval. When participants made a response or no response in 3,000 ms, the letter array was replaced by a buffer screen display for 1,000 ms. A schematic representation of the procedure is shown in Figure 1B. All participants were required to focus their gaze on the white fixation when the target appeared and respond to the target as quickly and accurately as possible, while ignoring pictures of faces that appeared in the left or right visual field.

### EEG recording and preprocessing

EEG data were recorded using a NeuroScan 64 Ag/AgCl electrode system, following the location and labeling of the extended 10–20 system. EEG data were amplified by a NeuroScan SynAmps 2 amplifier with a 0.025–100 Hz bandpass filter and a sampling frequency of 1,000 Hz. All electrode impedances were kept below 5 kΩ. Horizontal eye movements were monitored by placing HEOG electrodes laterally on the outer canthi of the eyes.

EEG data offline analyses were performed with a custom-made script supported by EEGLAB (Delorme and Makeig, 2004) in MATLAB (R2021b, The MathWorks, Inc.). Continuous EEG data were first down-sampled to 500 Hz and then digitally filtered using a cutoff of 0.1–30 Hz (roll-off 12 dB/octave). Subsequently, the data were further segmented into epochs from 200 ms before the start of the target presentation to 1,000 ms after the target presentation, and the average value in the 200 ms time window preceding stimulus onset was used for baseline correction. All instances of error and extreme epochs (RTs < 200 ms or > 2,000 ms) corresponding to behavioral exclusion criteria were discarded. Then, eye blinks, muscle artifacts, or other types of noise were removed from the data by independent component analysis (Delorme and Makeig, 2004). For eye movement artifacts, because the ERP components of interest to this study were P1 (80–130 ms) and N170 (140–200 ms), trials that occurred with horizontal eye movement within 200 ms after the onset of stimuli were directly excluded to guarantee participants did not move their eye from the central fixation point. Finally, epochs exceeding ± 100 μV were excluded, the clear data were again confirmed via visual inspection, and EEG data were transformed to an average reference. The mean percentages of trials removed in each condition were as follows: low load with happy face distractor (deaf vs. hearing: 6.92 vs. 3.35%); low load with neutral face distractor (deaf vs. hearing: 6.39 vs. 3.68%); low load with angry face distractor (deaf vs. hearing: 6.93 vs. 4.58%); low load with no distractor (deaf vs. hearing: 6.66 vs. 3.33%); high load with happy face distractor (deaf vs. hearing: 2.23 vs. 7.14%); high load with neutral face distractor (deaf vs. hearing: 7.34 vs. 2.57%); high load with angry face distractor (deaf vs. hearing: 10.60 vs. 5.47%); and high load with no distractor (deaf vs. hearing: 9.58 vs. 5.15%).

### Behavioral and ERP data analysis

For behavioral data, incorrect trials and RTs < 200 ms or > 2,000 ms trials were first excluded. Then, mean RTs under each condition were analyzed using a 2 × 2 × 4 three-way repeated measures analysis of variance (ANOVA) with deafness (deaf/hearing) as the between-subject variable, load (low/high), and face (happy face/neutral face/angry face/no face) as within-subject variables.

Based on visual inspection of the grand-average ERP waveform and previous studies (Calvo et al., 2014a; Schindler et al., 2022), the region of interest (ROI) of P1 and N170 components were selected as occipito-temporal regions, including P7, PO7, P8, and PO8. Then, the baseline-to-peak amplitudes of P1 (80–130 ms) and N170 (140–200 ms) were extracted after averaging these electrodes. Finally, P1 and N170 were separately analyzed using 2 × 2 × 4 three-way repeated measures ANOVAs with deafness (deaf/hearing) as the between-subject variable, load (low/high), and face (happy face/neutral face/angry face/no face) as the within-subject variable.

Furthermore, according to previous studies, visual processing at the behavioral level in deaf individuals might reflect covariation of the cortical processing of stimuli (Bottari et al., 2011). Therefore, in this study, a correlation analysis was performed between the interference effects of RTs and subtle ERP responses, aiming to clarify whether covariation between behavioral performance and brain activities also existed in deaf individuals' attention to peripheral faces. Interference effects were calculated as the participants' RTs for the target presented with face distractors minus the target presented without face distractors. The corresponding ERP responses were calculated as the ERP response to the target presented without face distractors subtracted from the target presented with face distractors.

All statistical analyses were performed using SPSS, version 28.0, with a two-tailed significance level of 0.05 used for all statistical tests. All *p*-values were Greenhouse-Geisser corrected if necessary, and LSD corrections were used for multiple comparisons. A partial eta-squared () was computed to describe effect sizes.

## Results

### Behavioral data

**Figure 2:**
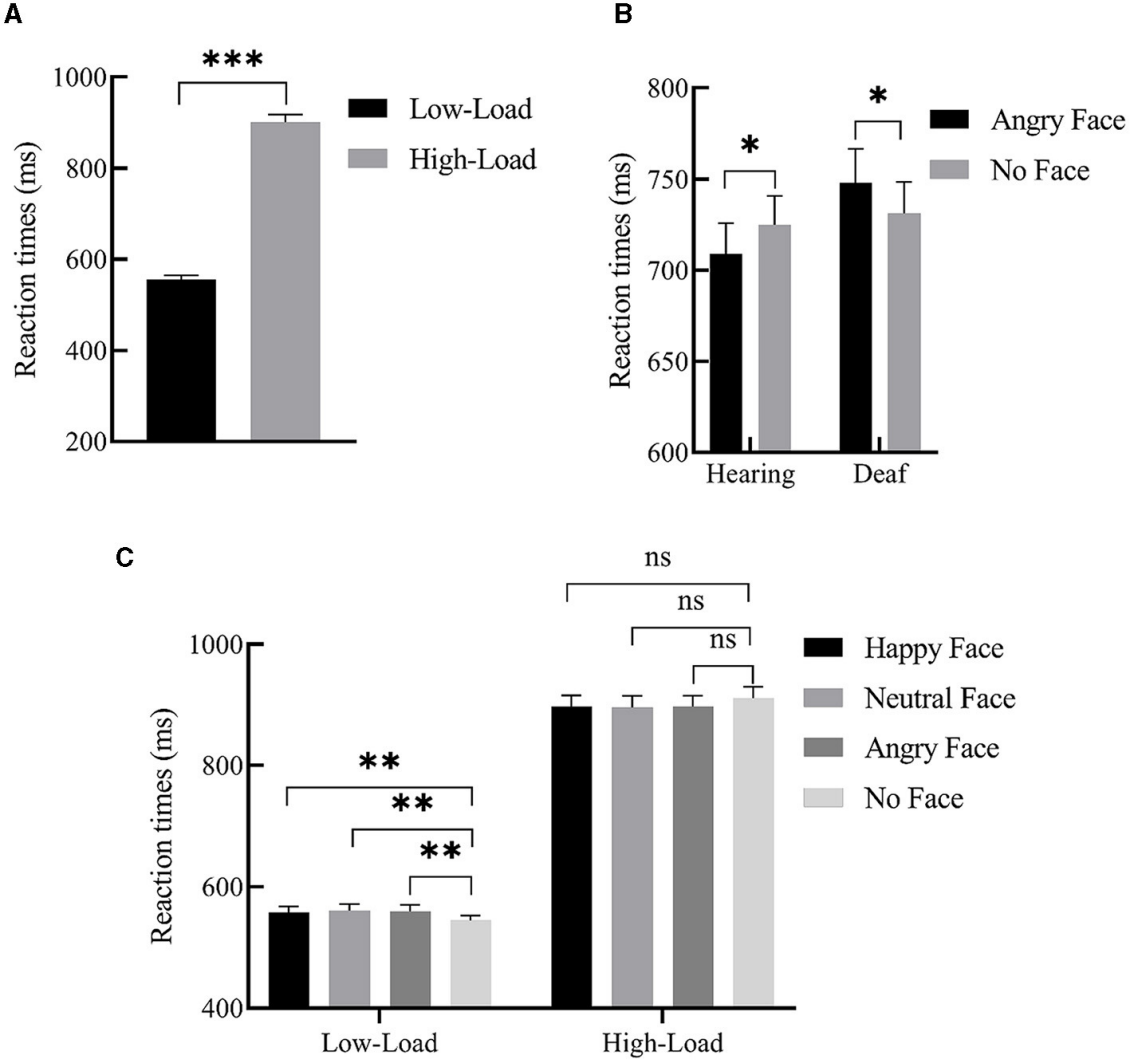
Significant main effects and interactions of RTs. **(A)** Main effect of load; participants' response was slower under high-load than low-load. **(B)** Interaction between deafness and face; angry faces slowed deaf participants' responses to the target, while facilitating hearing participants' responses (relative to no faces). **(C)** Interaction between load and face; under the low-perceptual load condition, peripheral happy, neutral, and angry face distractors produced interference effects, but no interference effect was found under high-perceptual load. Error bars represent the standard error. ***p < 0.001, **p < 0.01, *p < 0.05, ns, no significance.

For mean RTs, we performed 2 (deafness: deaf/hearing) × 2 (load: low/high) × 4 (face: happy face/neutral face/angry face/no face) repeated measures ANOVAs. There was a main effect of load, *F*_(1, 49)_ = 609.19, *p* < 0.001, $\eta_{p}^{2}$ = 0.93. *Post-hoc* analysis revealed that participants responded faster under low-perceptual load than under high-perceptual load (555.85 ± 9.33 ms vs. 900.35 ± 17.46 ms), indicating that our manipulation of the perceptual load was very effective. The two-way interaction between load and face was also significant, *F*_(3, 147)_ = 6.23, *p* = 0.001, $\eta_{p}^{2}$ = 0.29. Further simple effects analysis revealed that, only under the low-perceptual load condition, participants responded to the target presented with the peripheral happy (557.93 ± 9.79 ms, *p* < 0.01), neutral (560.90 ± 1 0.28 ms, *p* = 0.001), and angry faces (559.90 ± 10.52 ms, *p* < 0.01) more slowly than when faces were not present (544.69 ± 8.22 ms). In other words, the interference effect from faces was observed, but no interference effect was found under high perceptual load (*p* > 0.05). Importantly, the interaction of deafness by face reached significance, *F*_(3, 147)_ = 3.44, *p* = 0.02, $\eta_{p}^{2}$ = 0.18. A simple effects analysis revealed that deaf participants responded to the target presented with angry faces slower than when faces were not present (748.12 ± 19.49 ms vs. 731.13 ± 18.18 ms, *p* < 0.05); conversely, hearing participants responded to the target presented with angry faces faster than when faces were not present (709.06 ± 16.77 vs. 725.02 ± 15.81 ms, *p* < 0.05). In other words, angry faces slowed deaf participants' responses to the target while facilitating hearing participants' responses. The main effects of both deafness (*p* = 0.41) and face (*p* = 0.99) were not significant. A three-way interaction of deafness by load by face was also not found (*p* = 0.82). The significant effects of RTs are shown in Figure 2, and the mean RTs for target and interference effects under each condition in the hearing and deaf groups are shown in Table 3.

**Table 3:** The mean reaction times for central target/interference effects in each condition for two groups.

**Group**	**Load**	**Central target/interference effects (ms)**			
		**Happy face**	**Neutral face**	**Angry face**	**No face**
Hearing	Low	549.70 ± 63.55/11.33	552.54 ± 67.42/14.17	537.82 ± 63.18/-0.55	538.37 ± 53.77
	High	888.64 ± 141.05/-23.03	885.30 ± 135.51/-26.37	880.29 ± 142.47/-31.38	911.67 ± 140.10
Deaf	Low	566.16 ± 76.39/15.15	569.25 ± 79.42/18.24	581.97 ± 86.84/30.97	551.01 ± 63.64
	High	905.68 ± 120.16/-5.57	905.73 ± 141.23/-5.52	914.27 ± 108.13/3.02	911.25 ± 115.15

The mean reaction times (RTs) were presented as M ± SD. The interference effects were calculated as the participants' RTs for the target presented with face distractors minus the target presented without face distractors.

### ERP data

**Figure 3:**
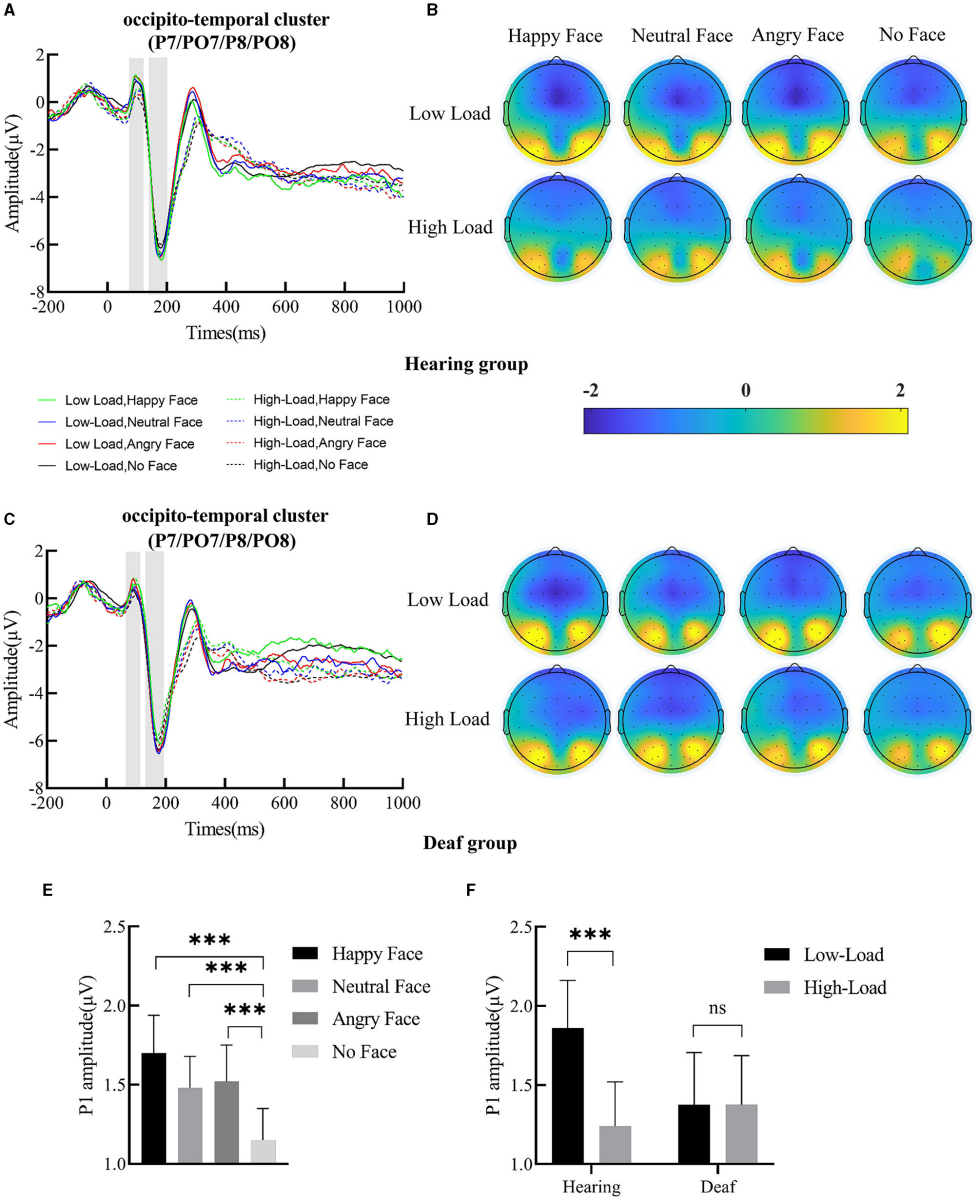
**(A)** Grand-averaged waveforms of P1 and N170 over occipito-temporal clusters (P7, PO7, P8, and PO8) in the hearing group; the shallow gray rectangular boxes represent the measure time window of P1 and N170. **(B)** Scalp topographic map of P1 under each condition for the hearing group. **(C)** Grand-averaged waveforms of P1 and N170 over the occipito-temporal cluster in the deaf group. **(D)** Scalp topographic map of P1 under each condition for the deaf group. **(E)** Main effect of face; the P1 amplitude for happy, angry, and neutral face distractors was larger than for no face distractors. **(F)** Interaction between deafness and load; only for the hearing group, the P1 amplitude decreased with increased central perceptual load. Error bars represent the standard error. ***p < 0.001, ns, no significance.

**Figure 4:**
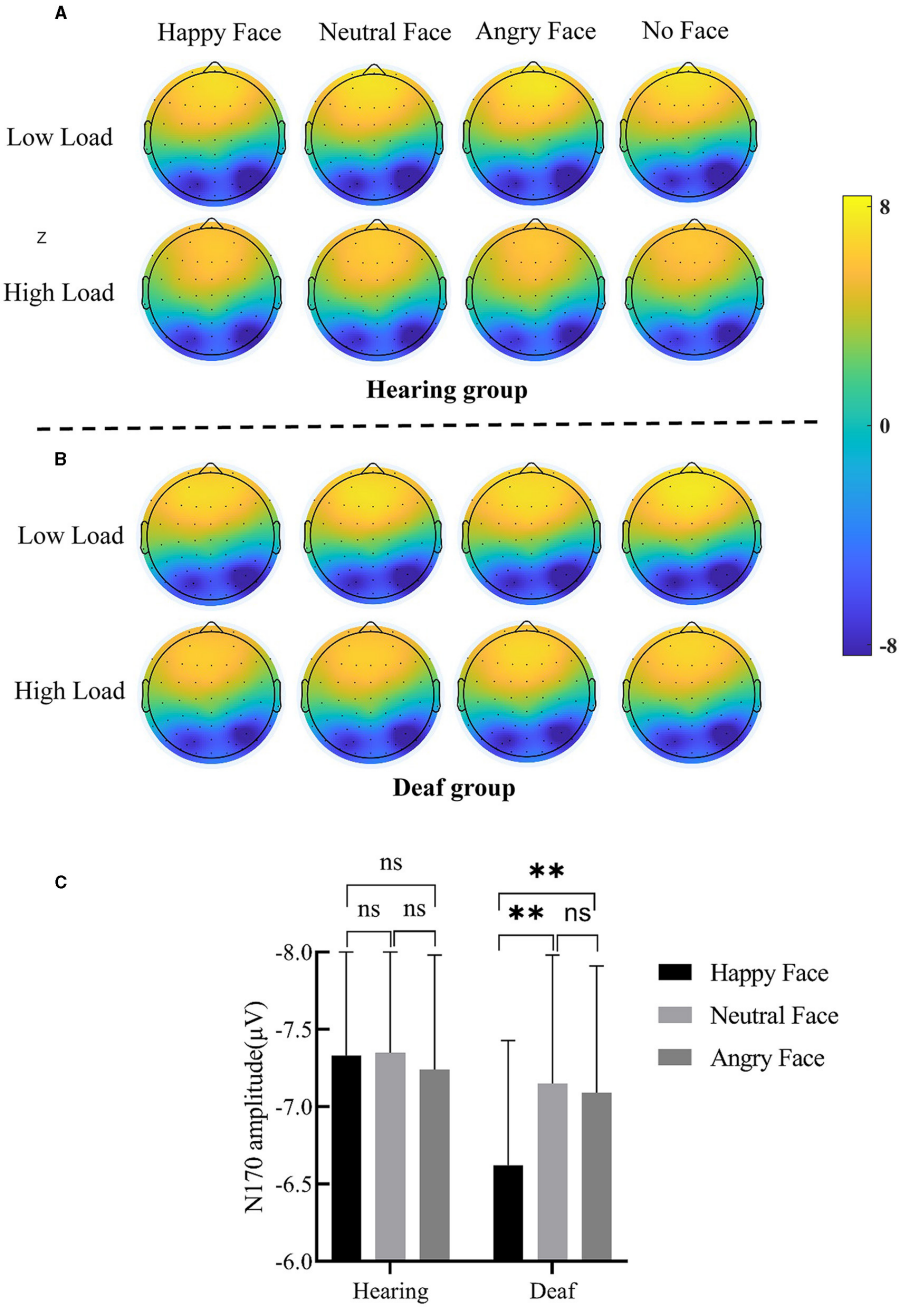
**(A)** Scalp topographic map of N170 under each condition for the hearing group. **(B)** Scalp topographic map of N170 under each condition for the deaf group. **(C)** Interaction between deafness and face; only for the deaf group, the N170 amplitude for angry and neutral face distractors was larger than happy face distractors. Error bars represent the standard error. **p < 0.01, ns, no significance.

The ERP waveforms and scalp topographic map of P1 and N170 under each condition in deaf and hearing groups are shown in Figures 3A–D, 4A–B.

#### P1

P1 amplitude yielded a main effect of load, *F*_(1, 49)_ = 7.11, *p* = 0.01, $\eta_{p}^{2}$ = 0.13; *post-hoc* comparisons revealed that the P1 amplitude under low-perceptual load (1.62 ± 0.22 μV) was larger than under high-perceptual load (1.31 ± 0.21 μV). A main effect of face was also found (Figure 3E), *F*_(3, 147)_ = 8.59, *p* < 0.001, $\eta_{p}^{2}$ = 0.15. Targets presented with happy (1.70 ± 0.24 μV*, p* < 0.001), neutral (1.48 ± 0.20 μV, *p* < 0.001), and angry (1.52 ± 0.23 μV, *p* < 0.001) faces elicited a higher P1 amplitude than targets presented without faces (1.15 ± 0.20 μV). The interaction between deafness and load was significant (Figure 3F), *F*_(1, 49)_ = 7.08, *p* = 0.01, $\eta_{p}^{2}$ = 0.13. Further simple effects analysis indicated that, for deaf individuals, P1 amplitude was not modulated by perceptual load (low vs. high = 1.38 ± 0.33 vs. 1.38 ± 0.31 μV, *p* > 0.05); however, for hearing individuals, P1 amplitude was reduced with perceptual load increase (low vs. high = 1.86 ± 0.30 vs. 1.24 ± 0.28 μV, *p* < 0.001). The main effect of deafness (*p* = 0.67), the two-way interaction of deafness by face (*p* = 0.67), and the three-way interaction of deafness by load by face (*p* = 0.95) did not reach significance.

#### N170

A main effect of face was discovered, *F*_(3, 147)_ = 5.60, *p* = 0.001, $\eta_{p}^{2}$ = 0.10. The N170 amplitude for neutral (−7.25 ± 0.56 μV, *p* < 0.001) and angry (−7.17 ± 0.55 μV, *p* < 0.01) faces was larger than that for no faces (−6.77 ± 0.53 μV). The N170 amplitude for happy faces (−6.98 ± 0.55) was also larger than for no faces, but only at a trending level (*p* = 0.10). The two-way interaction of deafness and face reached significance (Figure 4C), *F*_(3, 147)_ = 3.83, *p* = 0.01, $\eta_{p}^{2}$ = 0.07. Separating out the interaction showed that, for deaf participants, happy faces (−6.62 ± 0.81 μV) elicited a smaller N170 amplitude than neutral (−7.15 ± 0.783 μV, *p* < 0.01) and angry faces (−7.09 ± 0.82 μV, *p* < 0.01); however, for hearing participants, happy (−7.33 ± 0.73 μV, *p* < 0.001), neutral (−7.35 ± 0.75 μV, *p* < 0.001), and angry (−7.24 ± 0.74 μV, *p* < 0.01) faces produced comparable N170 amplitudes that were larger than no faces (−6.70 ± 0.71 μV). The main effects of load (*p* = 0.10) and deafness (*p* = 0.83), as well as the two-way interactions of deafness by load (*p* = 0.53), load by face (*p* = 0.86), and the three-way interaction of deafness by load by face, did not reach significance (*p* = 0.94).

### Correlation analysis

At the behavioral level, we found interference effects only existed in the condition of low perceptual load. To clarify the association between interference effects and ERP responses to face distractors under low perceptual load, a Pearson correlation between interference effects and ERP differential amplitudes under the same condition was computed.

**Figure 5:**
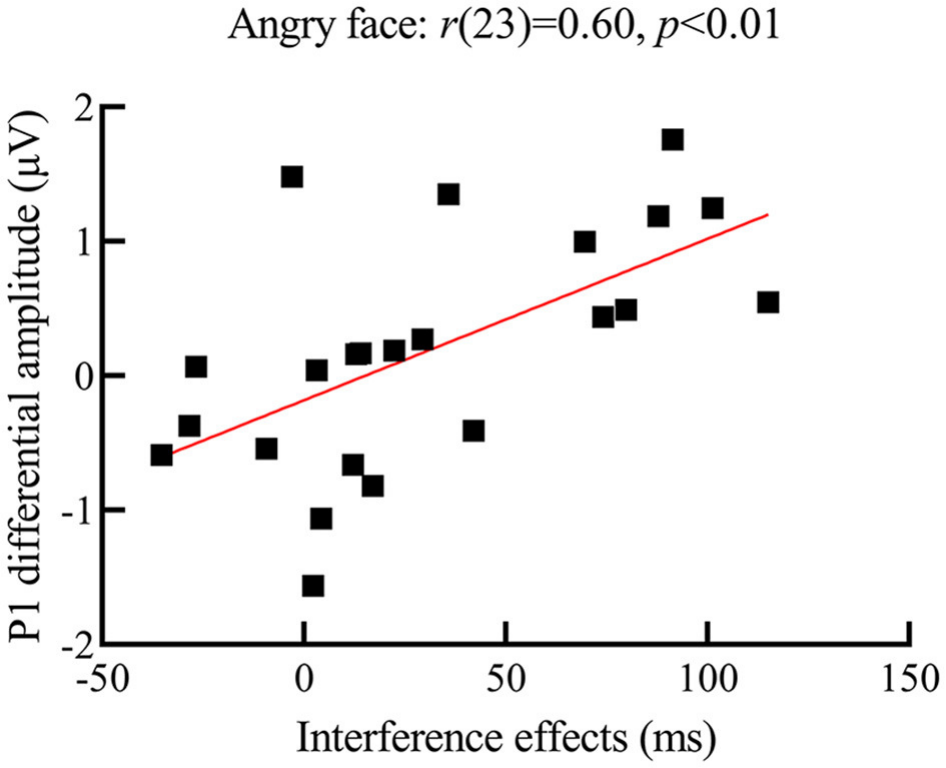
Scatterplot of correlation between interference effects and P1 differential amplitude for angry faces in the deaf group; the y-axis represents the P1 differential amplitude (i.e., P1 amplitude for the angry face distractors minus no face distractors); the x-axis represents the interference effect (i.e., participants' RTs for the target presented with face distractors minus face distractors not present); the larger the P1 differential amplitude for angry faces, the larger the interference effect from them.

Correlation analysis results showed that, for deaf participants, the P1 differential amplitude for angry faces was positively related to its interference effects (see Figure 5 for a scatterplot of the data), *r*(23) = 0.60, *p* < 0.01, indicating that the bigger the P1 differential amplitude for angry faces, the larger the interference effects from them. However, similar relationships were not found for happy and neutral faces [happy face: *r*(23) = 0.20, *p* = 0.35; neutral face: *r*(23) = −0.02, *p* = 0.92]. Furthermore, no relationships between N170 differential amplitude and interference effects from the face were found.

For hearing participants, no significant relationship between interference effects and ERP differential amplitudes was found. The complete Pearson correlation coefficients between the ERP differential amplitudes and interference effects from faces in deaf and hearing groups are shown in Table 4.

**Table 4:** The Pearson correlation coefficient between the ERP differential amplitude and interference effects from face distractors.

**Group**	**ERP differential amplitude**	**Pearson correlation coefficient (** ***r*** **)**		
		**Happy face**	**Neutral face**	**Angry face**
Deaf	P1	0.2	−0.02	0.60^**^
	N170	0.16	0.24	0.38
Hearing	P1	−0.28	−0.08	−0.32
	N170	−0.14	0.23	0.03

^**^p < 0.01.

## Discussion

Several previous studies have demonstrated that deaf individuals exhibit superior detection of peripheral motion and static abstract stimuli (e.g., geometrical shapes) at both behavioral and neural levels relative to hearing people (Neville and Lawson, 1987; Proksch and Bavelier, 2002; Bavelier et al., 2006). However, evidence on whether this advantage of deaf people is maintained for highly meaningful stimuli is scarce. In this study, a modified perceptual load paradigm was combined with an ERP technique to investigate the attention to peripheral facial expressions under central perceptual load in deaf and hearing individuals. Behaviorally, overall, participants' responses to the target were slower under high than low perceptual load, and interference effects from face distractors were eliminated by increasing the perceptual load. These results indicated that our manipulation of the perceptual load was highly successful. In addition, we found that only the interference effects from angry faces dissociated between deaf and hearing individuals. Specifically, angry faces slowed deaf participants' responses to the target while facilitating hearing participants' responses. For ERP data, there were four main findings. First, the P1 amplitude for targets presented with faces was larger than for targets presented without faces, suggesting that the peripheral faces did automatically capture participants' attention. Second, modulation of P1 amplitude by central load was found only in hearing individuals. Third, for deaf individuals, larger interference from angry face distractors was associated with higher P1 differential amplitude (angry faces minus no faces). Fourth, we observed that the N170 amplitude for happy face distractors was smaller than that for angry and neutral face distractors in the deaf group.

At the behavioral level, interference effects from face distractors were discovered under low perceptual load, but eliminated under high perceptual load. This was consistent with perceptual load theory, which suggests that visual processing is a capacity-limited process; when perceptual load is increased, early selective attention to distractors is reduced or even eliminated (Lavie, 1995, 2005). One intriguing finding was that angry faces slowed deaf participants' responses to the target while facilitating hearing participants' responses; yet, no similar effects for happy or neutral faces were observed. Angry faces represent a type of human hostility of the beholder, a kind of threat signal throughout evolution, and thus could preferentially capture individuals' attention compared with non-emotional stimuli (Öhman et al., 2001). When an angry face distractor is presented with letters, it automatically competes for cognitive resources with the letter, distracting participants' responses. However, in this study, participants were required to respond to the target as quickly and accurately as possible. Therefore, when the perceptual load at the center was high, hearing individuals may have actively inhibited spatial attention to the non-target location and focused attention on searching for the central target in order to fulfill the instruction. Their response under high perceptual load supports this interpretation, as they responded faster to the target presented with happy and neutral faces than when no distractors were present, though this effect did not reach significance. This may reflect a general inhibition of spatial attention to peripheral locations in these individuals.

However, significant interference effects from angry faces were observed in deaf individuals and appear to not be affected by available attentional resources. These results demonstrate that, although peripheral free attentional capacity may be limited, peripheral, task-irrelevant angry faces may be so salient to deaf individuals' awareness that it is difficult to inhibit attention to them. In a similar vein, Shalev et al. (2020) found that the perception of peripheral fearful faces that were also salient stimuli signaling threat was more tolerant to increasing eccentricities in deaf individuals compared to hearing individuals. This result is thought to reflect a compensatory mechanism following auditory deprivation, as the visual system of deaf individuals develops a higher sensitivity to visually substitutive stimuli representing dangerous signals (Shalev et al., 2020). This may also be a possible explanation for why threatening stimuli were such high-priority stimuli for deaf individuals that they could not ignore.

P1 is an exogenous component that generates from the extrastriatal cortex (Di Russo et al., 2002), indexing the stage of coarse sensory processing. Its amplitude is also sensitive to the salience of stimuli; for example, facial stimuli typically elicit a larger P1 than nonface objects (Rossion et al., 2003; Herrmann et al., 2005). Therefore, convergent with evidence from previous studies, the enlarged P1 amplitude for faces compared with no faces observed in this study suggests that peripheral faces can automatically capture participants' attention due to their visual salience, even though they were not relevant for the task. A more critical finding for P1 is that its amplitude for faces decreased with increasing load in hearing rather than deaf individuals. Prior ERPs and neuroimaging studies in the hearing population have reported similar modulation of P1-related neural activities for peripheral distractors by central perceptual load (Handy et al., 2001; Schwartz et al., 2005). For example, Schwartz et al. (2005) found that, as the load in the center increased, activity in the visual cortex related to irrelevant peripheral stimuli decreased. Therefore, we propose that the present P1 data in hearing individuals reflects the process of targets and peripheral faces competing for cognitive resources, which is consistent with the claim that the selective attention to peripheral goal-irrelevant stimuli is influenced by load at a very early stage (Lavie, 2005). Furthermore, this result is in line with a report by Hauthal et al. (2012) that showed that attention to peripheral face stimuli in deaf individuals is more resistant to central load increases when compared to hearing individuals. The relative immunity to central load in deaf individuals has been attributed to an enlarged sensory representation of the peripheral field in the primary visual cortex (Bavelier et al., 2006). This proposal is supported by an fMRI study conducted by Smittenaar et al. (2016), which indicated that deafness may result in greater structural and functional plasticity in the earliest stages of the primary visual cortex. Specifically, this study performed widefield population receptive field (pRF) mapping of the primary visual cortex in participants and discovered that the pRF encoding the peripheral visual field was larger in deaf participants than in hearing participants. Therefore, in this study, plastic changes in the primary visual cortex may explain the relative resistance to central load of the P1 response to peripheral faces in deaf individuals.

Furthermore, the P1 differential amplitude for angry faces (but not happy or neutral faces) was positively related to interference effects only in deaf individuals, indicating the more attention captured by angry faces, the more interference they produced. Indeed, the relationship between behavioral performance for detecting peripheral stimuli and early P1 amplitude in deaf individuals has been reported in previous research (Bottari et al., 2011). In the study by Bottari et al. (2011), participants were required to respond to peripheral target stimuli while keeping their head and eyes oriented toward a central fixation point. This study reported that larger P1 amplitudes were associated with shorter RTs for peripheral target stimuli in deaf participants but not in hearing participants, which was thought to reflect a co-variation between modified brain activity (cortical plasticity) and behavioral enhancement in deaf individuals. Therefore, the current correlation analysis results may imply that deaf individuals' attention to angry faces is supported by changes in neural activity in specific brain regions. However, this should be confirmed by neuroimaging techniques with higher spatial resolution, given the low spatial resolution limitation of ERPs. Interestingly, both the behavioral data and correlation analysis showed a special role for angry faces in differentiating deaf and hearing groups. This could imply that, in certain aspects, angry faces are more salient for deaf individuals than for hearing individuals. In our opinion, two possible aspects should be considered. First, the visual characteristics of angry faces and the method by which deaf individuals typically view static faces may specifically drive this effect. It has been demonstrated that when viewing static face pictures, deaf people looked at the eyes more frequently than the nose, whereas this was reversed in hearing people (Watanabe et al., 2011). The salient features of angry faces are precisely in the eye area, such as an inverted eyebrow shape (Fox et al., 2000). Therefore, deaf people may find it more difficult to inhibit their attention to angry faces because of conspicuous eyebrow features. Second, the intrinsic threatening value of angry faces may be more important for deaf individuals than hearing individuals because of the absence of auditory input. However, contrary to our prediction, we did not observe a different P1 amplitude between angry faces and neutral or happy faces. This result may indicate that, even for deaf individuals with greater peripheral attentional capacity, the early rapid discrimination of different emotional content in peripheral faces in the primary visual cortex was hindered when attentional resources were limited.

We found that, for deaf individuals, angry and neutral face distractors elicited higher N170 than happy face distractors. However, there were no similar emotional effects of N170 in hearing individuals. N170 is a face-sensitive ERP component, reflecting the process of structural coding of faces. Recently, an increasing number of studies have reported that N170 amplitude is modulated by facial expressions; for example, N170 for angry, fearful, disgusted, and happy faces was found to be larger than that for neutral faces (Batty and Taylor, 2003; Blau et al., 2007; Hinojosa et al., 2015). However, it has also been reported that the emotional effects of N170 were reduced or even abolished when attention was directed away from the face in hearing individuals (Eimer et al., 2003; Holmes et al., 2003). This finding was further confirmed by the current N170 results of hearing individuals in our sample. In our opinion, the unexpected emotional effects of N170 observed in deaf individuals may represent a successive process of coarse classification of threatening and non-threatening faces after happy, neutral, and angry faces capture attention (represented by P1). It has been demonstrated that when faces are presented in the periphery, happy faces are more easily recognized due to the higher visual saliency and diagnostic value of the smiling mouth (Calvo et al., 2014b). Therefore, the smaller N170 for happy faces observed in deaf individuals may suggest that they expend less attentional resources to judge happy faces as non-threat stimuli. This process may be driven by top-bottom attention in deaf individuals in an effort to avoid missing threat stimuli due to the absence of auditory input.

## Limitations

There were several limitations in this study that should be noted. First, in this study, static facial expressions were used as peripheral distractors. However, facial expressions are a highly dynamic phenomenon, making the static picture very unrealistic for participants. Especially for deaf people, dynamic facial expressions are critical semantic and grammatical components in sign language (Elliott and Jacobs, 2013). Furthermore, it has been suggested that the dynamic nature of facial expressions can facilitate recognition and enhance the intensity and arousal of facial emotion (Krumhuber et al., 2013). Therefore, whether deaf people exhibit the same advantage over hearing individuals in detecting angry faces when static expressions are replaced by dynamic expressions should be further studied. Second, we are aware that other confounding factors, apart from deafness, should be considered to explain the attention to angry faces observed in deaf individuals. Specifically, many studies have reported that deaf individuals experience more anxiety disorders than hearing individuals (Ushalnagar et al., 2019), which may lead to attentional bias toward threatening information (Bar-Haim et al., 2007). Therefore, in the future, when using threatening faces as a peripheral distractor, the anxiety factor should be controlled by matching the anxiety level between deaf and hearing samples. Finally, although our sample size was relatively larger than those used in similar studies, it was still a small sample size study. Therefore, a larger sample size should be used in the future to ensure that these effects in deaf individuals are solid.

## Conclusion

The present data demonstrate that, despite being under central perceptual load, deaf individuals exhibit less attentional inhibition toward peripheral goal-irrelevant angry faces than hearing individuals. Furthermore, they appear to discriminate between peripheral non-threatening and threatening faces during the stage of structural encoding of faces (140–200 ms). These results may reflect a compensatory mechanism in which, in the absence of auditory alertness to danger, the detection of visually threatening information outside of the current attentional focus has a high priority. In the future, confounding variables, such as anxiety, should be controlled, and the neural basis for the detection of angry faces in deaf individuals should be elucidated using other neuroimaging techniques, thereby enhancing our understanding of neural plasticity.

## Data availability statement

The raw data supporting the conclusions of this article will be made available by the authors, without undue reservation.

## Ethics statement

The studies involving humans were approved by the Chongqing Key Laboratory of Psychological Diagnosis and Education Technology for Children with Special Needs Research Ethics Board. The studies were conducted in accordance with the local legislation and institutional requirements. The participants provided their written informed consent to participate in this study. No potentially identifiable images or data are presented in this study.

## Author contributions

LY conceived and designed the experiments with the help of TW. JH took responsibility for the data processing and statistical analysis, visualization, and drafting of the whole manuscript. LY, JH, KL, YL, and LD carried out experiments. TW provided the critical idea for the revision of the manuscript for important intellectual content. All authors contributed to the article and approved the submitted version.
